# Combined Climate and Chemical Stressors: How Spatial Variability Shapes the Response of *Ficopomatus enigmaticus* (Fauvel, 1923) to Dimethyl Sulfoxide (DMSO) and Heatwaves, and What It Means for Ecotoxicology

**DOI:** 10.3390/jox15060181

**Published:** 2025-11-01

**Authors:** Verdiana Vellani, Manuela Piccardo, Francesca Provenza, Serena Anselmi, Valentina Pitacco, Lovrenc Lipej, Stanislao Bevilacqua, Monia Renzi

**Affiliations:** 1 Department of Life Sciences, University of Trieste, 34127 Trieste, Italy; verdiana.vellani@phd.units.it (V.V.); manuela.piccardo@units.it (M.P.); mrenzi@units.it (M.R.); 2 National Interuniversity Consortium for Marine Sciences (CoNISMa), 00196 Rome, Italy; 3 Bioscience Research Center, Via Aurelia Vecchia, 32, 58015 Orbetello, Italy; serena.anselmi@bsrc.it; 4 Marine Biology Station Piran, National Institute of Biology, Fornače 41, 6330 Piran, Slovenia; valentina.pitacco@nib.si (V.P.); lovrenc.lipej@nib.si (L.L.)

**Keywords:** wild populations, biomarkers, heat stress, combined stress, population responses

## Abstract

*Ficopomatus enigmaticus*, a reef-forming serpulid, has emerged as a promising candidate for biomonitoring and ecotoxicology studies. Recent research has focused on adult stress responses, highlighting the need to understand population-specific responses. This study employed a multi-biomarker approach to investigate how *F. enigmaticus* adults from two populations in the NE Adriatic (Site A) and NE Tyrrhenian (Site B) responded to chronic exposure to heat and chemical stress (dimethyl sulfoxide, DMSO), individually and in combination. The analysis detected significant differences in protein content and the activities of superoxide dismutase (SOD) and glutathione S-transferase (GST) between populations. Notably, no oxidative damage (measured as lipid peroxidation, LPO) was detected in any population or treatment. Similarly, no significant differences were detected in the integrated biomarker response index (IBRv2i). However, lower IBRv2i values at Site A suggested reduced stress conditions, possibly indicating that this site may have lower baseline stress. Overall, treatment effects were limited and site-specific: only the combined heat and DMSO exposure at Site A lowered GST activity compared to heat stress alone. Nevertheless, both populations exhibited broadly similar biochemical response patterns to stress. Our findings deepen the understanding of stress physiology in *F. enigmaticus*, underscoring the ecological importance of multi-stressor approaches in environmental monitoring.

## 1. Introduction

The Australian tubeworm, *Ficopomatus enigmaticus* (Fauvel, 1923), is a serpulid polychaete that builds and inhabits calcareous tubes in brackish and marine waters [1,2]. Although this species has a type locality in European waters, in an artificial canal opening into the English Channel [3], its sudden appearance in well-investigated environments suggests that it represents a newly introduced species [4,5]. As a fouling organism colonizing ship hulls, buoys, and port structures, this species has the potential to spread through maritime traffic [6,7] in temperate waters of both hemispheres [2,6,8]. Therefore, although *F. enigmaticus* currently has an almost cosmopolitan distribution, it is considered a non-indigenous species in European waters ([5] and references therein). Its native range is commonly believed to be the Southern part of Australia, but it has spread so widely and been established for so long that now it is not clear which was its original range [9]. Starting from single tubes, it can form larger aggregations and build reef-like structures up to three meters high in conditions ranging from mesohaline (8–18 PSU) to hypersaline (>40 PSU) [10]. Such reefs can span tens of square meters and exceed one meter in thickness [1,11]. In Tuscany (Italy), colonies of up to 150,000 individuals per square meter have been observed [1,2]. The high fecundity of this species favors its rapid numerical growth, as it can reproduce in different seasons [6,10,12]. *F. enigmaticus* is a suspension feeder that feeds on phytoplankton, zooplankton, and detrital particles, which it filters from the water using the cilia on its radioles [13]. By removing suspended particulate matter and contaminants, it can significantly contribute to purifying and increasing the transparency of the water [14,15,16]. *F. enigmaticus* is also a bioconstructor [17], building reefs that increase the three-dimensionality of the environment, stabilize local conditions, modulate essential ecosystem processes, and accumulate organic matter that serves as a trophic resource for other organisms, promoting biodiversity [18,19]. However, its excessive proliferation may interfere with human activities and infrastructures, and the high filtering capacity can also have negative effects, modifying water quality and habitat structure, and consequently altering indigenous communities [20].

Due to its characteristics, which include ease of sampling and identification, as well as the presence of gametes available in different seasons, the species has been proposed as a potential model for monitoring marine and brackish waters [21,22]. Building on this potential, *F. enigmaticus* has been used in ecotoxicological research, particularly in the application of biomarkers related to oxidative stress in polychaetes. Research has mainly focused on using larvae and spermatozoa in ecotoxicological tests [21,23,24,25], while studies involving adult specimens are more limited [26,27,28,29,30,31]. Even fewer studies have addressed chronic exposure (28 days) in adults [28,29]. Only two of those studies have examined the effects of climate change on this bioconstructor [26,30]. Only one study analyzed the enzymatic response to chemical pollutants in combination with heat waves caused by climate change [30]. The results of the enzymatic activity tests on adult organisms demonstrated that both adults and embryos are valid model organisms for toxicity tests, as they are sensitive enough to detect various types of stress, including that caused by increased temperature [30]. Investigations into the combined effects of warming and pollution have emerged as a major research theme, given that aquatic invertebrates are highly vulnerable to both stressors [32,33]. Indeed, the combined exposure of *F. enigmaticus* to contaminants (e.g., copper) and heatwaves has been found to have synergistic effects compared to exposure to single stressors [30]. In their natural environments, organisms experience multiple concurrent anthropogenic stressors, which can interact additively or multiplicatively, often resulting in effects greater than those caused by individual stressors alone [34,35]. Nevertheless, the way organisms respond to combinations involving less toxic but widespread agents, such as laboratory solvents, remains to be clarified.

Dimethyl sulfoxide (DMSO) is a commonly used cosolvent in ecotoxicological testing due to its ability to dissolve both polar and nonpolar substances, facilitating their permeability through biological membranes without causing significant damage [36,37,38,39]. It is particularly useful for testing hydrophobic toxicants on aquatic organisms, as these require solvents that ensure adequate solubility and bioavailability [40,41,42]. While the Organization for Economic Co-operation and Development [43] recommends a maximum concentration of 100 mg/L (0.1 mL/L), concentrations of up to 10% (*v*/*v*) are often used in practice to effectively solubilize lipophilic substances [44]. Several recent studies have examined the tolerance of dimethyl sulfoxide (DMSO) in the context of aquatic toxicity with model species. For example, Hedge et al. [45] found that concentrations of up to 1% (*v*/*v*) DMSO showed negligible toxicity in the embryos of the zebrafish *Danio rerio* Hamilton, 1822. Furthermore, *Ceriodaphnia dubia* Richard, 1894 appears to be sensitive to DMSO exposure due to the high mortality rate recorded, suggesting that appropriate concentrations for this species should remain below 0.5% [46]. In general, other species, such as the freshwater crustacean *Daphnia magna* Straus, 1820, the marine crustaceans *Artemia franciscana* Kellogg, 1906, and *Allorchestes compressa* Dana, 1852, have also been shown to experience effects following exposure to DMSO at concentrations of 0.1–10% [44]. While most studies have examined the effects of DMSO upon acute exposure, chronic testing reveals more subtle sublethal effects [47]. Furthermore, the effects on adult organisms are yet to be clarified, as well as the response in combination with thermal stress.

The effects of toxicants on wild populations are inherently context-dependent because biotic and abiotic conditions in their native habitats critically influence their responses [48]. This ecological reality presents a significant challenge in ecotoxicology: a species’ sensitivity to toxicants is greatly affected by its population origin, yet comparative data remain scarce [49]. Most toxicological knowledge comes from monospecific laboratory bioassays [50], which are simplified systems that cannot replicate the environmental complexity of natural ecosystems (e.g., fluctuating abiotic conditions and species interactions) where wild populations often exhibit lower toxicant sensitivity compared to their laboratory-reared counterparts [49]. This suggests that standard bioassays may systematically overestimate environmental risks. These findings reveal a critical limitation of current risk assessment paradigms: artificial laboratory conditions poorly predict real-world ecological outcomes. Furthermore, it is crucial to better understand how populations from different habitats respond to toxicants, since site-specific environmental conditions may drive population-level adaptations affecting stress susceptibility.

This study aimed to evaluate how adults of the model species, *F. enigmaticus*, from different populations respond to chronic chemical and heat stress, both individually and in combination. The effects were examined in terms of energy reserves, the activity of enzymes involved in the first and second lines of defense, and oxidative stress damage. A multi-biomarker approach was adopted to investigate the total protein content (PROT), superoxide dismutase (SOD), glutathione S-transferase (GST), and lipid peroxidation (LPO) in these reef-forming polychaetes collected from two distinct Mediterranean ecoregions.

## 2. Materials and Methods

### 2.1. Experimental Setup, Animal Collection, and Exposure

This study was conducted in collaboration between two research centers (University of Trieste and Bioscience Research Center), following a jointly developed, standardized protocol aimed at ensuring consistency in animal husbandry and exposure procedures across laboratories. Specifically, both laboratories followed the same protocol, which included acclimatization and exposure in standardized tanks under identical conditions. In detail, *Ficopomatus enigmaticus* reefs were collected in spring 2023 from two different sites (specifically, within one week at the end of May in both sites): one in an ephemeral coastal brackish pond in Koper, Slovenia (Site A, NE Adriatic Sea), and one in the Orbetello Lagoon in Tuscany, Italy (Site B, NE Tyrrhenian Sea). At the time of sampling, the two sites were characterized by distinct environmental conditions: Site A, salinity 15‰, dissolved oxygen 40%, temperature 22 °C, pH 8.2, depth 0.1 m; Site B, salinity 28‰, dissolved oxygen 78%, temperature 21 °C, pH 7.8, depth 0.3 m. Briefly, Site A represents a small, shallow, and transient coastal pond with low salinity and limited spatial extent (a few tens of meters), where *F. enigmaticus* occurs in scattered patches. This site has not been documented in the existing literature. In contrast, Site B, the Orbetello Lagoon, is a well-characterized, historically impacted Mediterranean coastal system with documented occurrences of eutrophication, anoxic events, and chemical contamination in which *F. enigmaticus* has been present for decades [51,52,53].

**Figure 1:**
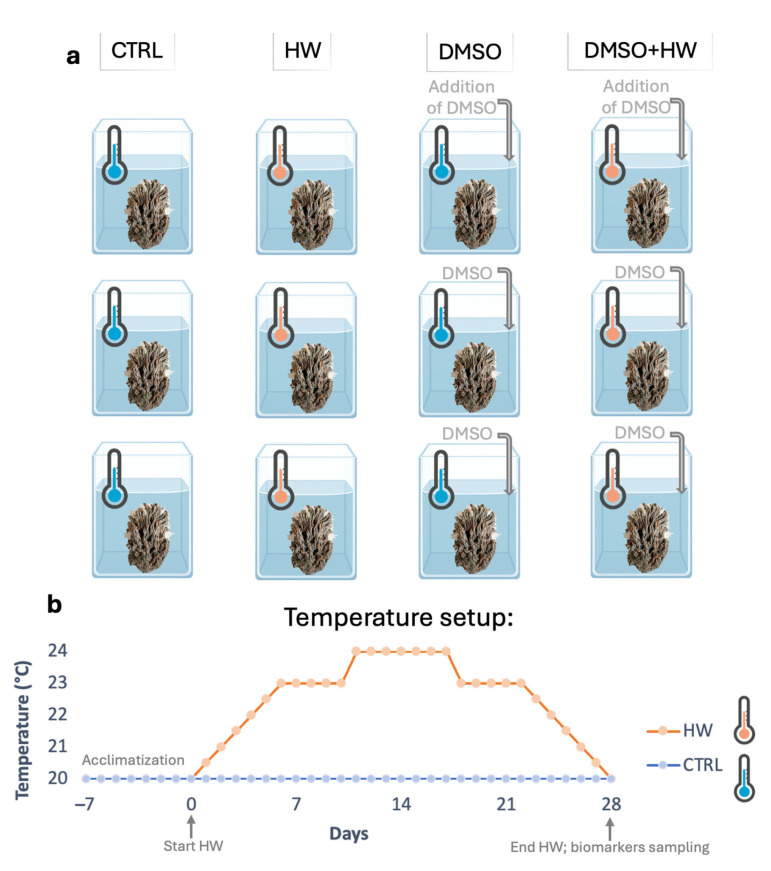
(**a**) A schematic representation of the experimental design including four conditions and three replicates per condition: control (CTRL); heatwave (HW); 0.5% dimethyl sulfoxide (DMSO) exposure; and the combined HW + DMSO treatment. (**b**) Temperature increase and decrease ramp for treatments involving HW. All operations were performed according to the same acclimatization and exposure protocol in both research centers involved. The onset of the experiments of exposure was synchronous between the two laboratories.

Following collection, the samples were transported in semi-humid and shaded conditions to separate laboratories within one hour: the University of Trieste for the Site A samples and the Bioscience Research Center (BsRC) for the Site B samples. Upon arrival at the laboratories, colonies containing sediment or associated fauna in their interstitial spaces were gently rinsed with seawater, fragmented into smaller portions, rinsed again, and then distributed into twelve 1 L aquaria containing 0.45 µm filtered seawater (FSW). The following acclimatization lasted 1 week under the following conditions: 15‰ salinity, 20 ± 1 °C, 12:12 h light/dark cycle (light maintained with AKKEE LED Aquarium Light, 12 V, Shenzhen, Guangdong, China), and constant aeration. Specimens were fed three times a week with an algal suspension of *Isochrysis galbana* Parke (Prymnesiophyceae) from internal propagation, achieving a final concentration of 1 × 10^4^ cells/mL per aquarium. After acclimatization, the organisms were transferred to the experimental setup shown in Figure 1a. The experimental setup consisted of three replicates for each of the four conditions: control (same experimental conditions as during acclimatization), heatwave (HW), DMSO exposure, and a combined treatment involving both stressors (DMSO + HW). Total water changes were made weekly during the exposure period. Feeding, lighting, and aeration conditions were maintained at the same levels as those used during acclimatization. The temperature ramp, shown in Figure 1b, was performed over 28 days as follows: starting at 20 °C, the temperature was increased by 0.5 °C per day until reaching 23 °C, which was then maintained for 5 days. It was then raised to 24 °C and maintained for 7 days. This process was then reversed using the same method, intensity, and timing as the initial increase. The experiment was conducted in coordination with the two research centers. The start and end times were coordinated and standardized, and all the procedures were carried out during the light phase of the photoperiod.

DMSO (Sigma-Aldrich, Merck KGaA, Darmstadt, Germany; CAS No. 67-68-5) was chosen as the chemical stressor at a final concentration of 0.5% because model organisms in ecotoxicology studies have exhibited various responses to this substance, some of which were adverse [44]. Thus, it is crucial to determine how the proposed model species will respond to this widely used ecotoxicological compound.

At the end of the exposure period, the individuals were extracted from the calcareous tubes and placed into Falcon tubes (approximately 1 g per replicate, around 80 individuals), forming pools of organisms to reduce inter-individual variability related to sex or age differences. The Falcon was then placed at −80 °C for subsequent biomarker analysis.

### 2.2. Biomarker Analysis

Biomarkers of oxidative stress and damage, including total protein content (PROT), SOD, GST, and LPO, were measured at the end of the exposure period. To ensure the reliability of the data obtained, all biomarker measurements were performed at a single, ACCREDIA-certified laboratory [54], using standardized protocols across all samples, as follows.

Each *F. enigmaticus* pool was analyzed in duplicate, and the results were expressed as the mean ± SD. Each pool represented one of three replicates per experimental condition (*n* = 3).

To collect the cellular fraction (S9) according to the method described by De Marchi et al. [55], animal pools were thawed and homogenized using an Ultra-Turrax, with ice baths to maintain sample temperature. Homogenates were extracted in 50 mM K-phosphate buffer (pH 7.5) containing 2 mM EDTA, at a sample-to-buffer ratio of 1:4 *w*/*v* [56,57]. The samples were ultracentrifuged (Centrifuge 5910 R, Eppendorf AG, Hamburg, Germany) at 15,000× *g* for 15 min at 4 °C. The obtained supernatant (S9 fraction) was aliquoted and stored at −20 °C until biomarker analysis.

#### 2.2.1. Total Protein Content (PROT)

The extracted cellular fraction (S9) was subjected to protein quantification using the method described by Lowry et al. [58]. This colorimetric assay is based on reactions involving copper (CuSO_4_·5H_2_O), Folin–Ciocalteu reagent, and 0.5 M NaOH, which together produce a blue-color complex. The intensity of the resulting color is measured at 750 nm using a plate reader (LTEK INNO, LTEK, Seongnam, Republic of Korea) with a final volume of 200 µL per well. Bovine serum albumin (BSA) was used as a standard, in the range of 0–500 µg/mL. Protein concentrations were expressed in mg/mL. Enzyme activity was then normalized to the total protein content [58,59].

#### 2.2.2. Superoxide Dismutase (SOD)

SOD activity was measured using the indirect method based on the enzyme’s ability to inhibit pyrogallol autoxidation, as described by Gao et al. [60]. The previously obtained S9 fraction was directly assayed in a 96-well plate (final volume of 200 µL per well) by adding pyrogallol and a Tris-EDTA buffer solution (pH 8.2). The reaction was allowed to proceed for three minutes before measuring the absorbance at 420 nm using an LTEK INNO plate reader (LTEK, Seongnam, Republic of Korea). The results are reported in units (U) per mg of protein, where one unit is defined as the amount of enzyme required to cause 50% inhibition of pyrogallol autoxidation.

#### 2.2.3. Glutathione S-Transferase (GST)

GST activity was quantified in the S9 fraction using the method described by Habig et al. [61]. This enzyme catalyzes the conjugation of the substrate 1-chloro-2,4-dinitrobenzene (CDNB, 60 mM) and reduced glutathione (GSH, 10 mM). The samples were mixed directly in a 96-well plate with a reaction solution containing 0.1 M phosphate buffer (pH of 6.5), GSH, and CDNB. Absorbance kinetics were measured at 340 nm for five minutes using an LTEK INNO plate reader (LTEK, Seongnam, Republic of Korea), with a final volume of 200 µL per well. The change in absorbance was used to calculate GST activity based on the extinction coefficient for CDNB (ε = 9.6 mM^−1^ cm^−1^). The results were normalized to protein content and time and expressed as µmol min^−1^ µg^−1^.

#### 2.2.4. Lipid Peroxidation (LPO)

The LPO level was determined by quantifying malondialdehyde (MDA) using the method described by Mihara & Uchiyama [62]. A solution containing 0.6% (*w*/*v*) thiobarbituric acid and 1% (*v*/*v*) phosphoric acid was added to the aliquots of the S9 fraction. The samples were heated to 96 °C for 25 min in an oven, then cooled and centrifuged with 1-butanol at 4000 rpm for five minutes at room temperature (Centrifuge 5910 R, Eppendorf AG, Hamburg, Germany). The MDA content in the supernatant was measured spectrophotometrically in a 1 cm path-length cuvette, by reading the absorbance difference between 535 and 520 nm using a C-7100 spectrophotometer (Peak Instruments, Shanghai, China). Results were expressed as U/mg of protein.

### 2.3. IBRv2i Index

To summarize and simplify the interpretation of biomarker responses, the ‘Integrated Biological Responses version 2i’ index (IBRv2i), unweighted, was utilized, providing an overall assessment of the organism’s health status. The IBRv2i index was calculated following the method described by Mattos et al. [63]. Briefly, it is an index that sums the absolute individual variations in the biomarkers of each animal, and it is based on the concept of a ‘control group’ reference deviation to establish the normal or basal levels of the biomarkers. The magnitude of the index values can be interpreted as the impact of the treatments on organisms: high index values indicate poor health status or more stressed organisms.

### 2.4. Data Analysis and Statistics

The biomarkers and IBRv2i data were checked for normality using the Shapiro–Wilk test and for homogeneity of variance using Levene’s test with R software version 4.3.3 [64], combined with RStudio version 2025.05.0+496 [65]. As both assumptions were respected for all variables, a two-way ANOVA was performed on untransformed data to test for differences in the response of organisms to treatments between sites, followed by a Tukey post hoc test if required. Then, Eta Squared (η^2^) was calculated using the formula: η^2^ = SS factor/(SS factor + SS residuals), to represent the variance explained by a factor in ANOVA [66,67]. Effect sizes were estimated using eta-squared (η^2^) for each factor in the analysis of variance (ANOVA). According to the guidelines of Cohen [68] and Lakens [69], the interpretation of η^2^ is as follows: negligible (η^2^ < 0.01), small (η^2^ between 0.01 and 0.06), medium (η^2^ between 0.06 and 0.14), and large (η^2^ ≥ 0.14).

## 3. Results

### 3.1. Biomarker Responses

**Figure 2:**
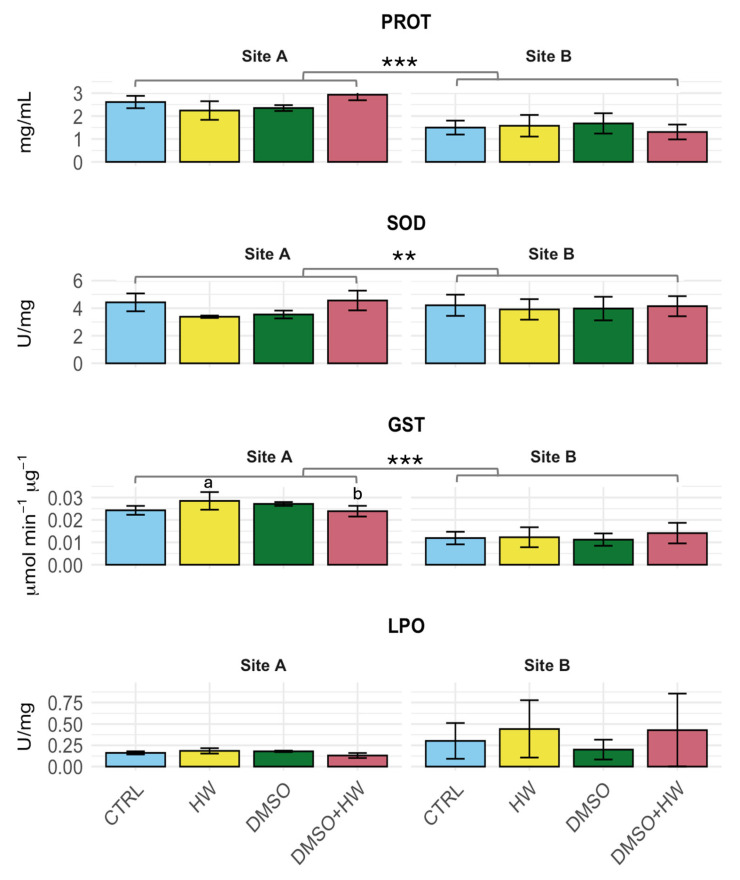
Mean values (±SD; *n* = 3) of the response of four biomarkers—protein content (PROT), superoxide dismutase (SOD), glutathione S-transferase (GST), and lipid peroxidation (LPO)—were compared between Site A (NE Adriatic) and Site B (NE Tyrrhenian) under four treatments: control (CTRL), heat wave (HW), dimethyl sulfoxide (DMSO), and combined stress (DMSO + HW). Different letters indicate statistically significant differences between treatments within a site, while asterisks denote significant differences between sites as follows: *p* < 0.01 (**) and *p* < 0.001 (***).

No significant effects of treatments were detected for PROT, SOD, and LPO (Table 1, Figure 2). Significant variations between sites were detected for PROT and SOD, with large effect sizes (η^2^ = 0.74 and η^2^ = 14, respectively). Specifically, Site A showed substantially higher concentrations of protein content (2.6 ± 0.3 mg/mL) than Site B (1.5 ± 0.4 mg/mL), whereas SOD activity revealed minor variations between sites (3.97 ± 0.69 vs. 4.06 ± 0.75 U/mg for Site A and B, respectively) (Table 1, Figure 2). For LPO, the analysis did not highlight significant variations between sites (Table 1), although a moderate effect size (η^2^ = 0.09) was recorded and the inspection of graph in Figure 2 suggested higher values for Site B associated with a higher heterogeneity of responses, which could have masked possible differences between the two sites for this variable.

For GST, ANOVA detected a significant ‘Treatment × Site’ interaction and a moderate effect size (η^2^ = 0.09), indicating that differences among treatments varied between the two sites. While the treatments did not differ from each other at Site B, the heatwave exposure (HW) at Site A resulted in significantly higher GST activity (0.028 ± 0.04 µmol min^−1^ µg^−1^) than the combined stress condition (DMSO + HW: 0.024 ± 0.002 µmol min^−1^ µg^−1^; *p* = 0.028). The overall GST activity in all the conditions at Site A, however, showed higher values than at Site B, as also indicated by the large proportion of variance explained by the eta-squared (η^2^ = 0.58).

### 3.2. IBRv2i

**Figure 3:**
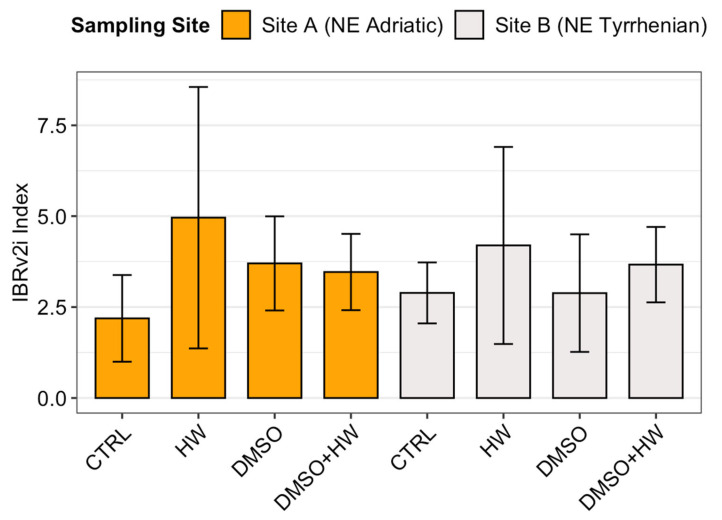
Mean IBRv2i index values (±SD; *n* = 3) demonstrating differential stress responses between two wild populations from Site A (NE Adriatic) vs. Site B (NE Tyrrhenian) in the four treatments: control (CTRL), heat wave (HW), dimethyl sulfoxide (DMSO), and combined stress (DMSO + HW).

No statistically significant differences in IBRv2i values were found between sites or among treatments (Table 1). However, in most cases (i.e., CTRL, DMSO, and DMSO + HW) the mean IBRv2i values at Site A (2.19, 3.70, and 3.46, respectively) were lower than those at Site B (2.89, 2.88, and 3.67), suggesting a general lower stress condition (Figure 3). In contrast, the heat wave treatment (HW) appeared to have a greater impact on the population from Site A, which showed a higher IBRv2i value (4.96) compared to Site B (4.19). Furthermore, it is worth noting that, in general, treatments showed higher IBRv2i values than the control at their respective sites, except CTRL and DMSO at Site B, where no apparent differences were observed. Although the results were not statistically significant (*p* = 0.348), the large η^2^ value (0.18) suggested a potential effect of treatment on the overall biomarker profile.

## 4. Discussion

*Ficopomatus enigmaticus* is a widespread, reef-building species well suited for biomonitoring and ecotoxicology studies [21,22]. While initial studies focused on larval and sperm toxicity [21,23], recent research has shifted to adult responses under chemical and climate-related stressors [27,29,30]. This emphasizes the need to evaluate population-specific sensitivities, for which biomarkers are essential early warning tools in environmental monitoring [70,71]. In this context, analyzing the biomarkers of two populations of *F. enigmaticus* provided insight into their baseline physiological responses to different environments.

The observed differences in protein content between the two populations may reflect underlying environmental stress. The generally lower values detected in Site B could be related to the environmental characteristics and/or pollution level to which the organisms were subjected. This likely resulted in a high metabolic cost and, consequently, a high cost of energy reserves invested in survival rather than growth and reproduction [30,72,73]. This energy conservation theory, proposed by Sokolova [74] and by Sokolova et al. [73], suggests that organisms exposed to chronic environmental stressors adopt low-energy defense strategies. The Orbetello Lagoon (Tuscany, Italy) has faced multiple environmental crises over the years, including recurrent fish mortality events, eutrophication, chemical contamination, and anoxic episodes [51]. The population at site B may have been affected by these stressful conditions, as it has recorded a lower total protein quantity. Site A is also exposed to severe environmental conditions in summer, including sharp temperature, salinity, and oxygen availability oscillations. Nevertheless, sampling was conducted in spring, when environmental conditions at Site A may have been relatively favorable. However, despite the basal difference in this biomarker, the two populations exhibited similar behaviors in response to stress, with no change in protein content under the stress conditions tested compared to the controls.

Despite a significant site effect in SOD activity, with Site B exhibiting higher levels of SOD, the similarity in control values across sites suggests population-level homeostasis under baseline conditions. Few articles address the differences in biomarkers between sites, and the limited literature on the topic is inconclusive. Reported patterns of spatial variability for SOD are inconsistent and appear to depend on various biological and environmental factors, as well as species [75,76]. Differences in SOD were found among sites for the serpulid reef-former *Sabellaria alveolata* Linnaeus, 1767, revealing a latitudinal gradient in Atlantic Europe from northwest England to central Portugal [77]. The results of the study conducted by Curd and colleagues [77] showed that SOD followed an ‘abundant edge’ pattern in their latitudinal distribution, with generally higher levels in the northern or southern edges of the species’ area of distribution than in the center. The three poleward sites examined by Curd and colleagues stood out from the other sites due to their elevated metabolic activity levels, suggesting that environmental conditions at these sites were more physiologically stressful for this species [77]. SOD is often used as a biomarker and is considered a sensitive indicator of oxidative stress in response to various stressors in polychaetes [78,79,80]. However, this study did not detect differences between treatments. In particular, regarding the effects of increased temperature, the tested HW does not appear to impact this species with oxidative stress or damage, a finding also reported by Vellani et al. [30] with a different HW for the same exposure time, and for both the only HW condition and a combined condition with metal (i.e., copper) and heat stress. Our results support once again the energy conservation theory linked to low-energy defense strategies, such as maintaining non-inducible levels of protective molecules like antioxidant enzymes. This approach minimizes the energetic cost of continuously upregulating responses like SOD, enabling organisms to conserve energy for vital processes such as growth and reproduction.

Although the GST activity of the exposed organisms did not differ from control levels, we observed significantly higher values under the HW condition compared to combined stress exposure (DMSO + HW). This trend aligns with heat stress responses documented in other bioconstructors, such as tropical corals, which exhibit increased GST activity with temperature increases of +4 °C [81]. In the non-serpulid polychaete *Hediste (Nereis) diversicolor* (O.F. Müller, 1776), an increase in temperature of +5 °C nearly doubles GST levels [82]. Moreover, the freshwater bivalve *Anodonta cygnea* Linnaeus, 1758 exhibits similar GST upregulation under warming conditions [83]. However, GST activity shows variable behavior toward temperature across species and contexts [84,85]. These parallels may indicate that GST induction represents a conserved (though not universal) stress response in aquatic invertebrates [86]. In particular, the GST attenuation under combined chemical and heat stress revealed in our study highlights the complexity of multi-stress interactions [87], where concomitant stressors can supplant standard thermal response models.

Our study found no significant differences in levels of LPO or the first line of antioxidant defense, such as SOD, among treatments. This could be explained by the increased activity of GST, a phase II biotransformation enzyme. According to Dias et al. [81,88], the increased GST activity could counterbalance the harmful effects of reactive oxygen species (ROS) on lipids. Average LPO levels were higher in Site B with respect to Site A in all treatments, although no significant differences were detected between sites. On one hand, this could be due to the limited number of samples and the high heterogeneity among samples. However, only Site B showed a high variability in LPO levels, suggesting intraspecific variations in LPO as a result of higher and more heterogeneous levels of oxidative stress characterizing this site.

The results of the present study reveal both similarities and differences compared to the work of Vellani et al. [30] on a different Tuscan *F. enigmaticus* population. They conducted their experiment with two brief, intense marine heatwaves separated by a brief recovery period. In contrast, this study tested a single, moderate, prolonged heat stress, with a slightly lower cumulative temperature. Despite the difference in thermal regimes, both studies observed remarkable stability in SOD and LPO across all treatments. This suggests that these oxidative stress markers are robustly conserved in *F. enigmaticus*, regardless of population origin. This study and that of Vellani et al. [30] documented comparable GST induction during heat stress events. This indicates a threshold response to temperatures exceeding 24–25 °C, which appears independent of heating duration or recovery intervals. However, the populations diverged in their response to combined stressors: Vellani et al. [30] study found that, compared to the control group, the sample exposed to heat waves and copper showed increased GST activity. In contrast, in our case, the population exposed to heat stress combined with DMSO displayed lower GST levels compared to the heat wave (HW) condition. This difference likely reflects the distinct chemical properties of the co-stressors: copper’s pro-oxidant nature appears to synergize with heat stress, driving enhanced detoxification responses [30], while DMSO’s radical-scavenging capacity [89,90] may buffer against oxidative damage during combined stress conditions. Thanks to its antioxidant properties, DMSO seems to reduce the negative effects of increased temperature in *F. enigmaticus*, although this remains a speculative interpretation. This suggestion offers intriguing prospects for the future of conservation efforts involving reef-building species, despite the need for further clarification on the use of DMSO for this purpose. Concluding the comparison, it is also worth observing that Vellani et al. employed a broader multi-biomarker approach (with 10 biomarkers in total), possibly increasing sensitivity to sublethal effects. This suggests that future studies should expand the biomarker panel to improve the resolution of integrative stress assessments. Furthermore, although copper did not elicit a detectable effect in their study, more research is needed to better understand how *F. enigmaticus* responds to different classes of pollutants, both individually and in combination.

Biomarker analyses of several benthic species have revealed site-specific physiological responses associated with varying degrees of environmental contamination. For instance, *H. diversicolor* from polluted areas, such as the Seine estuary or the Oued Souss, exhibited elevated GST activity and reduced acetylcholinesterase (AChE) and catalase levels, as well as increased LPO [91,92]. Similar responses have been observed in estuaries under moderate anthropogenic pressure [93] and in another polychaete species, *Perinereis gualpensis* Jeldes, 1963, from sites contaminated by metals and polycyclic aromatic hydrocarbons (PAHs) in Chile [94]. These variations indicate long-term adaptations to environmental stress. By analyzing how organisms from reference sites (representing pristine or minimally impacted environments) respond to pollutants versus how organisms from potentially polluted locations respond, researchers can establish baseline physiological ranges, quantify pollution-induced stress responses, and identify population-level adaptations to environmental contaminants [76,83,91,92,93,94,95]. Combining multiple biomarkers to create integrated stress profiles that reflect the cumulative impact of environmental pressures is a particularly powerful approach, as suggested by the European MSFD [96].

IBR analysis provides a broader overview of the physiological and biochemical conditions of the reef-forming species under study. Although no significant differences were detected between sites or treatments, the data showed a trend toward higher values in the HW treatment at both sites, which is consistent with greater physiological stress. This finding aligns with other references on *F. enigmaticus* and heat stress [30]. Generally, high IBR values have been recorded for other benthic species with increased temperature, including the brackish water clam *Corbicula japonica* Prime, 1864 [97], the saltwater clam *Cerastoderma edule* (Linnaeus, 1758) [98], the mussel *Mytella charruana* (A. d’Orbigny, 1846) [99], and the freshwater snail, *Bellamya bengalensis* (Lamarck, 1822) [100]. However, our results are contrary to what is usually found in combined conditions, where high IBR values are typically observed [30,99,101]. This could be caused by the presence of DMSO in the combined effect, which appears to result in an antioxidant effect. The high variability of the IBR data could be due to the small sample size (*n* = 3), which could have reduced the power of the analysis to detect significant effects of the treatments. However, as each replicate sample was a pool of a large number of individuals, it is likely that the observed variability may be the result of an intrinsic heterogeneity of response to stress among individuals in *F. enigmaticus*.

Altogether, our results suggest that DMSO at the tested concentration (0.5%) can be a suitable cosolvent for *F. enigmaticus* under laboratory conditions, since it did not impair the oxidative system of this species, but rather, it may buffer its responses to thermal and chemical stress. However, the potential attenuation of stress responses mediated by DMSO as a ‘buffering’ effect should be considered when interpreting biomarker responses to pollutants (e.g., GST activity), as it may modulate oxidative pathways. Although our study confirms the compatibility of *F. enigmaticus* adults with this solvent, caution is advised when using DMSO as a solubilizer for ecotoxicological and behavioral assays, as well as when interpreting the biochemical results.

Despite some site-specific differences likely driven by local environmental conditions and microevolutionary histories, both groups exhibited remarkably consistent biochemical patterns under heat and chemical stress, as well as under combined stress conditions. This convergence in stress physiology indicates a conserved response strategy across environments, which reinforces the reliability of using this species as a suitable model for ecotoxicological monitoring.

## 5. Conclusions

Our results demonstrate that *Ficopomatus enigmaticus* exhibits consistent responses to oxidative stress across populations despite site-specific basal differences. This finding supports the robustness of this species as a useful model organism in toxicological studies. The absence of oxidative damage under combined heat and stress conditions suggests that this bio-builder species employs energy-saving strategies to cope with chronic environmental pressures. From an ecotoxicological perspective, these results underscore the importance of using multiple biomarkers and integrated indices because single parameters may not capture sublethal effects. Furthermore, the modulatory response of DMSO highlights its suitability as a solvent under controlled laboratory conditions and emphasizes the need for caution when interpreting biomarker responses because its properties may mask stress induced by pollutants. In conclusion, this study highlights the need for more research on the stress ecology of this species. Specifically, the HW tested had a limited impact on the oxidative stress experienced by this species; however, greater effects were recorded in *F. enigmaticus*, according to literature reports. Similarly, the effects of combined chemical and thermal stress remain unclear. Future studies should expand the panel of biomarkers when applying integrative approaches and test how *F. enigmaticus* responds to metals and other chemical pollutants, both individually and in combination. Overall, *F. enigmaticus* emerges as a suitable model organism for biomonitoring and ecotoxicological studies involving multiple stressors across large-scale monitoring programs.

## Figures and Tables

**Table 1:** Results of the two-way ANOVA and Tukey post hoc tests for each biomarker and IBRv2i. Unless indicated otherwise, the post hoc Tukey test for both sites yielded the following results: CTRL = DMSO = HW = DMSO + HW.

	Source	d.f.	SS	MS	η^2^	F	*p*
PROT	Treatment	3	0.000022	0.000008	0.010	0.595	0.622
	Site	1	0.001661	0.0016613	0.740	132.035	0.000
	Treatment × Site	3	0.000064	0.0000212	0.030	1.687	0.185
	Residuals	40	0.000503	0.0000126			
SOD	Treatment	3	0.1958	0.0653	0.060	1.2	0.319
	Site	1	0.4618	0.4618	0.140	8.488	0.005
	Treatment × Site	3	0.1686	0.0562	0.050	1.033	0.386
	Residuals	52	2.8293	0.0544			
GST	Treatment	3	127,276	42,425	0.005	0.340	0.796
	Site	1	14,930,468	14,930,468	0.580	119.716	0.000
	Treatment × Site	3	2,247,608	749,203	0.090	6.007	0.001
	Residuals	52	6,485,213	124,716			
	Tukey post hoc						
	Site A:	CTRL = DMSO = DMSO + HW; CTRL = DMSO = HW; HW > DMSO + HW					
	Site B:	CTRL = DMSO = HW = DMSO + HW					
LPO	Treatment	3	2.305	0.7682	0.090	1.441	0.245
	Site	1	0.065	0.0653	0.003	0.123	0.728
	Treatment × Site	3	1.479	0.4929	0.060	0.924	0.438
	Residuals	40	21.33	0.5332			
IBRv2i	Treatment	3	12.79	4.263	0.180	1.182	0.348
	Site	1	0.17	0.173	0.002	0.048	0.829
	Treatment × Site	3	2.5	0.834	0.030	0.231	0.873
	Residuals	16	57.72	3.608			

## Data Availability

The original contributions presented in this study are included in the article/Supplementary Materials. Further inquiries can be directed to the corresponding authors.

## Supplementary Materials

The following supporting information can be downloaded at: https://www.mdpi.com/article/10.3390/jox15060181/s1, Table S1: Summary of mean ± standard deviation for all biomarkers, grouped by site and treatment conditions; Table S2: Mean values and standard deviations of IBRv2I across sites and treatments.

## References

[1] Bianchi C.N., Morri C. (1996). *Ficopomatus* ‘Reefs’ in the Po River Delta (Northern Adriatic): Their Constructional Dynamics, Biology, and Influences on the Brackish-Water Biota. Mar. Ecol..

[2] Bianchi C.N., Morri C. (2000). Marine Biodiversity of the Mediterranean Sea: Situation, Problems and Prospects for Future Research. Mar. Pollut. Bull..

[3] Fauvel P. (1923). Un Nouveau Serpulien d’eau Saumatre *Mercierella Ng Enigmatica* n. Sp. Bull. Soc. Zool. Fr..

[4] Cognetti G. (1954). Forme Della *Mercierella Enigmatica* Fauvel Nella Nuova Stazione Del Lago Di Patria. Ital. J. Zool..

[5] Langeneck J., Lezzi M., Pasqua M.D., Musco L., Gambi M.C., Castelli A., Giangrande A. (2020). Non-Indigenous Polychaetes along the Coasts of Italy: A Critical Review. Mediterr. Mar. Sci..

[6] Eno C., Clark R., Sanderson W. (1997). Non-Native Marine Species in British Waters: A Review and Directory. Jt. Nat. Conserv. Comm..

[7] Oliva M., De Marchi L., Vieira Sanches M., Pires A., Cuccaro A., Baratti M., Chiellini F., Morelli A., Freitas R., Pretti C. (2020). Atlantic and Mediterranean Populations of the Widespread Serpulid *Ficopomatus enigmaticus*: Developmental Responses to Carbon Nanotubes. Mar. Pollut. Bull..

[8] Ten Hove H.A., Kupriyanova E.K. (2009). Taxonomy of Serpulidae (Annelida, Polychaeta): The State of Affairs. Zootaxa.

[9] Styan C., McCluskey C., Sun Y., Kupriyanova E. (2017). Cryptic Sympatric Species across the Australian Range of the Global Estuarine Invader *Ficopomatus enigmaticus* (Fauvel, 1923) (Serpulidae, Annelida). Aquat. Invasions.

[10] Dittmann S., Rolston A., Benger S., Kupriyanova E. (2009). Habitat Requirements, Distribution and Colonisation of the Tubeworm Ficopomatus Enigmaticus in the Lower Lakes and Coorong.

[11] Fornós J.J., Forteza V., Martínez-Taberner A. (1997). Modern Polychaete Reefs in Western Mediterranean Lagoons: *Ficopomatus enigmaticus* (Fauvel) in the Albufera of Menorca, Balearic Islands. Palaeogeogr. Palaeoclimatol. Palaeoecol..

[12] Meadows P.S., Meadows A., Murray J.M.H. (2012). Biological Modifiers of Marine Benthic Seascapes: Their Role as Ecosystem Engineers. Geomorphology.

[13] Hille S., Kunz F., Markfort G., Ritzenhofen L., Zettler M. (2021). First Record of Mass Occurrence of the Tubeworm *Ficopomatus enigmaticus* (Fauvel, 1923) (Serpulidae: Polychaeta) in Coastal Waters of the Baltic Sea. BioInvasions Rec..

[14] Brundu G., Magni P. (2021). Context-Dependent Effect of Serpulid Reefs on the Variability of Soft-Bottom Macrobenthic Assemblages in Three Mediterranean Lagoons (Sardinia, Italy). Estuar. Coast. Shelf Sci..

[15] Davies B.R., Stuart V., de Villiers M. (1989). The Filtration Activity of a Serpulid Polychaete Population (*Ficopomatus enigmaticus* (Fauvel)) and Its Effects on Water Quality in a Coastal Marina. Estuar. Coast. Shelf Sci..

[16] Piccardo M., Vellani V., Anselmi S., Bentivoglio T., Provenza F., Renzi M., Bevilacqua S. (2024). The First Evidence of the Water Bioremediation Potential of *Ficopomatus enigmaticus* (Fauvel 1923): From Threat to Resource?. Water.

[17] Ellison A.M., Bank M.S., Clinton B.D., Colburn E.A., Elliott K., Ford C.R., Foster D.R., Kloeppel B.D., Knoepp J.D., Lovett G.M. (2005). Loss of Foundation Species: Consequences for the Structure and Dynamics of Forested Ecosystems. Front. Ecol. Environ. Ecol. Soc. Am..

[18] Bruschetti M., Bazterrica M., Fanjul E., Luppi T., Iribarne O. (2011). Effect of Biodeposition of an Invasive Polychaete on Organic Matter Content and Productivity of the Sediment in a Coastal Lagoon. J. Sea Res..

[19] Ingrosso G., Abbiati M., Badalamenti F., Bavestrello G., Belmonte G., Cannas R., Benedetti-Cecchi L., Bertolino M., Bevilacqua S., Bianchi C.N. (2018). Mediterranean Bioconstructions Along the Italian Coast. Adv. Mar. Biol..

[20] Katsanevakis S., Wallentinus I., Zenetos A., Leppäkoski E., Çinar M.E., Oztürk B., Grabowski M., Golani D., Cardoso A.C. (2014). Impacts of Invasive Alien Marine Species on Ecosystem Services and Biodiversity: A Pan-European Review. Aquat. Invasions.

[21] Oliva M., Mennillo E., Barbaglia M., Monni G., Tardelli F., Casu V., Pretti C. (2018). The Serpulid *Ficopomatus enigmaticus* (Fauvel, 1923) as Candidate Organisms for Ecotoxicological Assays in Brackish and Marine Waters. Ecotoxicol. Environ. Saf..

[22] Vellani V., Oliva M., Pretti C., Renzi M. (2025). Stress-Related Molecular Biomarkers to Monitor the Effects of Global Changes on Calcifying Reef-Forming Organisms: A Review in the Mediterranean. J. Mar. Sci. Eng..

[23] Cuccaro A., De Marchi L., Oliva M., Sanches M.V., Freitas R., Casu V., Monni G., Miragliotta V., Pretti C. (2021). Sperm Quality Assessment in *Ficopomatus enigmaticus* (Fauvel, 1923): Effects of Selected Organic and Inorganic Chemicals across Salinity Levels. Ecotoxicol. Environ. Saf..

[24] Oliva M., Manzini C., Bontà Pittaluga G., Kozinkova L., De Marchi L., Freitas R., Fabi G., Pretti C. (2019). *Ficopomatus enigmaticus* Larval Development Assay: An Application for Toxicity Assessment of Marine Sediments. Mar. Pollut. Bull..

[25] Vieira Sanches M., Oliva M., Pires A., De Marchi L., Cuccaro A., Freitas R., Baratti M., Pretti C. (2020). Relationship between Wild-Caught Organisms for Bioassays and Sampling Areas: Widespread Serpulid Early-Development Comparison between Two Distinct Populations after Trace Element Exposure. Ecotoxicol. Environ. Saf..

[26] Bezuidenhout M. (2021). The Implications of Climate Change for the Invasive Tube Worm *Ficopomatus enigmaticus*. Master’s Thesis.

[27] Casu V., Tardelli F., De Marchi L., Monni G., Cuccaro A., Oliva M., Freitas R., Pretti C. (2019). Soluble Esterases as Biomarkers of Neurotoxic Compounds in the Widespread Serpulid *Ficopomatus enigmaticus* (Fauvel, 1923). J. Environ. Sci. Health Part B.

[28] Cuccaro A., Oliva M., De Marchi L., Vieira Sanches M., Bontà Pittaluga G., Meucci V., Battaglia F., Puppi D., Freitas R., Pretti C. (2022). Biochemical Response of *Ficopomatus enigmaticus* Adults after Exposure to Organic and Inorganic UV Filters. Mar. Pollut. Bull..

[29] De Marchi L., Oliva M., Freitas R., Neto V., Figueira E., Chiellini F., Morelli A., Soares A.M.V.M., Pretti C. (2019). Toxicity Evaluation of Carboxylated Carbon Nanotubes to the Reef-Forming Tubeworm *Ficopomatus enigmaticus* (Fauvel, 1923). Mar. Environ. Res..

[30] Vellani V., Cuccaro A., Oliva M., Pretti C., Renzi M. (2024). Assessing Combined Effects of Long-Term Exposure to Copper and Marine Heatwaves on the Reef-Forming Serpulid *Ficopomatus enigmaticus* through a Biomarker Approach. Mar. Pollut. Bull..

[31] Zebral Y.D., da Silva Fonseca J., Marques J.A., Bianchini A. (2019). Carbonic Anhydrase as a Biomarker of Global and Local Impacts: Insights from Calcifying Animals. Int. J. Mol. Sci..

[32] Dinh K.V., Konestabo H.S., Borgå K., Hylland K., Macaulay S.J., Jackson M.C., Verheyen J., Stoks R. (2022). Interactive Effects of Warming and Pollutants on Marine and Freshwater Invertebrates. Curr. Pollut. Rep..

[33] Sokolova I., Lannig G. (2008). Interactive Effects of Metal Pollution and Temperature on Metabolism in Aquatic Ectotherms: Implications of Global Climate Change. Clim. Res..

[34] Halpern B.S., Walbridge S., Selkoe K.A., Kappel C.V., Micheli F., D’Agrosa C., Bruno J.F., Casey K.S., Ebert C., Fox H.E. (2008). A Global Map of Human Impact on Marine Ecosystems. Science.

[35] Halpern B.S., Frazier M., Potapenko J., Casey K.S., Koenig K., Longo C., Lowndes J.S., Rockwood R.C., Selig E.R., Selkoe K.A. (2015). Spatial and Temporal Changes in Cumulative Human Impacts on the World’s Ocean. Nat. Commun..

[36] Brayton C.F. (1986). Dimethyl Sulfoxide (DMSO): A Review. Cornell Vet..

[37] Sum A.K., Pablo J.J. (2003). de Molecular Simulation Study on the Influence of Dimethylsulfoxide on the Structure of Phospholipid Bilayers. Biophys. J..

[38] Szmant H.H. (1975). Physical Properties of Dimethyl Sulfoxide and Its Function in Biological Systems. Ann. N. Y. Acad. Sci..

[39] Williams A.C., Barry B.W. (2004). Penetration Enhancers. Adv. Drug Deliv. Rev..

[40] Di L., Kerns E.H. (2006). Biological Assay Challenges from Compound Solubility: Strategies for Bioassay Optimization. Drug Discov. Today.

[41] Modrzyński J.J., Christensen J.H., Brandt K.K. (2019). Evaluation of Dimethyl Sulfoxide (DMSO) as a Co-Solvent for Toxicity Testing of Hydrophobic Organic Compounds. Ecotoxicology.

[42] Stibany F., Ewald F., Miller I., Hollert H., Schäffer A. (2017). Improving the Reliability of Aquatic Toxicity Testing of Hydrophobic Chemicals via Equilibrium Passive Dosing–A Multiple Trophic Level Case Study on Bromochlorophene. Sci. Total Environ..

[43] OECD (2019). Guidance Document on Aquatic Toxicity Testing of Difficult Substances and Mixtures.

[44] Huang Y., Cartlidge R., Walpitagama M., Kaslin J., Campana O., Wlodkowic D. (2018). Unsuitable Use of DMSO for Assessing Behavioral Endpoints in Aquatic Model Species. Sci. Total Environ..

[45] Hedge J.M., Hunter D.L., Sanders E., Jarema K.A., Olin J.K., Britton K.N., Lowery M., Knapp B.R., Padilla S., Hill B.N. (2023). Influence of Methylene Blue or Dimethyl Sulfoxide on Larval Zebrafish Development and Behavior. Zebrafish.

[46] Bigi S., Schlappa K., Anselmi S., Provenza F., Renzi M. (2025). Uptake Through Feeding and/or Culture Medium of 0.5% Dimethyl Sulfoxide (DMSO): Biological Response of *Daphnia magna* and *Ceriodaphnia dubia* in Ecotoxicity Tests. Water.

[47] Stevens A.-S., Pirotte N., Plusquin M., Willems M., Neyens T., Artois T., Smeets K. (2015). Toxicity Profiles and Solvent–Toxicant Interference in the Planarian *Schmidtea mediterranea* after Dimethylsulfoxide (DMSO) Exposure. J. Appl. Toxicol..

[48] Thorp J.H., Gloss S.P. (1986). Field and Laboratory Tests on Acute Toxicity of Cadmium to Freshwater Crayfish. Bull. Environ. Contam. Toxicol..

[49] Romero-Blanco A., Alonso Á. (2022). Laboratory versus Wild Populations: The Importance of Population Origin in Aquatic Ecotoxicology. Environ. Sci. Pollut. Res..

[50] Brouwer A., Murk A.J., Koeman J.H. (1990). Biochemical and Physiological Approaches in Ecotoxicology. Funct. Ecol..

[51] Renzi M. (2022). La Laguna di Orbetello. Storia, Ambiente, Gestione e Progetti Futuri.

[52] Bombelli V., Lenzi M., Schramm W., Nienhuis P.H. (1996). Italy—The Orbetello Lagoon and the Tuscan Coast. Marine Benthic Vegetation: Recent Changes and the Effects of Eutrophication.

[53] Bianchi C.N., Morri C. (2001). The Battle Is Not to the Strong: Serpulid Reefs in the Lagoon of Orbetello (Tuscany, Italy). Estuar. Coast. Shelf Sci..

[54] (2018). General Requirements for the Competence of Testing and Calibration Laboratories 2018.

[55] De Marchi L., Neto V., Pretti C., Figueira E., Chiellini F., Soares A.M.V.M., Freitas R. (2017). Physiological and Biochemical Responses of Two Keystone Polychaete Species: *Diopatra neapolitana* and *Hediste diversicolor* to Multi-Walled Carbon Nanotubes. Environ. Res..

[56] Provenza F., Rampih D., Pignattelli S., Pastorino P., Barceló D., Prearo M., Specchiulli A., Renzi M. (2022). Mussel Watch Program for Microplastics in the Mediterranean Sea: Identification of Biomarkers of Exposure Using *Mytilus galloprovincialis*. Ecol. Indic..

[57] Vidal-Liñán L., Bellas J. (2013). Practical Procedures for Selected Biomarkers in Mussels, *Mytilus galloprovincialis*—Implications for Marine Pollution Monitoring. Sci. Total Environ..

[58] Lowry O.H., Rosebrough N.J., Farr A.L., Randall R.J. (1951). Protein Measurement with the Folin Phenol Reagent. J. Biol. Chem..

[59] DuBois M., Gilles K.A., Hamilton J.K., Rebers P.A., Smith F. (1956). Colorimetric Method for Determination of Sugars and Related Substances. Anal. Chem..

[60] Gao R., Yuan Z., Zhao Z., Gao X. (1998). Mechanism of Pyrogallol Autoxidation and Determination of Superoxide Dismutase Enzyme Activity. Bioelectrochem. Bioenerg..

[61] Habig W.H., Pabst M.J., Jakoby W.B. (1974). Glutathione S-Transferases. The First Enzymatic Step in Mercapturic Acid Formation. J. Biol. Chem..

[62] Mihara M., Uchiyama M. (1978). Determination of Malonaldehyde Precursor in Tissues by Thiobarbituric Acid Test. Anal. Biochem..

[63] Mattos J.J., Siebert M.N., Bainy A.C.D. (2024). Integrated Biomarker Responses: A Further Improvement of IBR and IBRv2 Indexes to Preserve Data Variability in Statistical Analyses. Environ. Sci. Pollut. Res..

[64] R Core Team (2024). R: A Language and Environment for Statistical Computing.

[65] Posit team RStudio: Integrated Development Environment for R 2025.05.0+496. https://posit.co/download/rstudio-desktop/.

[66] Cohen J. (1973). Eta-Squared and Partial Eta-Squared in Fixed Factor Anova Designs. Educ. Psychol. Meas..

[67] Pierce C.A., Block R.A., Aguinis H. (2004). Cautionary Note on Reporting Eta-Squared Values from Multifactor ANOVA Designs. Educ. Psychol. Meas..

[68] Cohen J. (1988). Statistical Power Analysis for the Behavioral Sciences.

[69] Lakens D. (2013). Calculating and Reporting Effect Sizes to Facilitate Cumulative Science: A Practical Primer for t-Tests and ANOVAs. Front. Psychol..

[70] Louis Y.D., Bhagooli R., Kenkel C.D., Baker A.C., Dyall S.D. (2017). Gene Expression Biomarkers of Heat Stress in Scleractinian Corals: Promises and Limitations. Comp. Biochem. Physiol. Part C Toxicol. Pharmacol..

[71] Monserrat J.M., Martínez P.E., Geracitano L.A., Lund Amado L., Martinez Gaspar Martins C., Lopes Leães Pinho G., Soares Chaves I., Ferreira-Cravo M., Ventura-Lima J., Bianchini A. (2007). Pollution Biomarkers in Estuarine Animals: Critical Review and New Perspectives. Comp. Biochem. Physiol. Toxicol. Pharmacol. CBP.

[72] Knochel A. (2017). The Effects of Thermal Stress on Fluorescent Protein Expression in an Indo-Pacific Scleractinian Coral Species, *Acropora tenuis*. Indep. Study Proj. ISP Collect..

[73] Sokolova I.M., Frederich M., Bagwe R., Lannig G., Sukhotin A.A. (2012). Energy Homeostasis as an Integrative Tool for Assessing Limits of Environmental Stress Tolerance in Aquatic Invertebrates. Mar. Environ. Res..

[74] Sokolova I.M. (2013). Energy-Limited Tolerance to Stress as a Conceptual Framework to Integrate the Effects of Multiple Stressors. Integr. Comp. Biol..

[75] Mishra D., Singh K.D., Singh A.K. (2022). A Study on Superoxide Dismutase Activity in a Freshwater Fish *Labeo rohita*: A Way of Assessing Aquatic Health. Eur. J. Biomed. Pharm. Sci..

[76] Manduzio H., Monsinjon T., Galap C., Leboulenger F., Rocher B. (2004). Seasonal Variations in Antioxidant Defences in Blue Mussels *Mytilus edulis* Collected from a Polluted Area: Major Contributions in Gills of an Inducible Isoform of Cu/Zn-Superoxide Dismutase and of Glutathione *S*-Transferase. Aquat. Toxicol..

[77] Curd A., Boyé A., Cordier C., Pernet F., Firth L.B., Bush L.E., Davies A.J., Lima F.P., Meneghesso C., Quéré C. (2021). Environmental Optima for an Ecosystem Engineer: A Multidisciplinary Trait-Based Approach. Sci. Rep..

[78] De Marchi L., Pretti C., Chiellini F., Morelli A., Neto V., Soares A.M.V.M., Figueira E., Freitas R. (2019). The Influence of Simulated Global Ocean Acidification on the Toxic Effects of Carbon Nanoparticles on Polychaetes. Sci. Total Environ..

[79] Freitas R., de Marchi L., Moreira A., Pestana J.L.T., Wrona F.J., Figueira E., Soares A.M.V.M. (2017). PPhysiological and Biochemical Impacts Induced by Mercury Pollution and Seawater Acidification in *Hediste diversicolor*. Sci. Total Environ..

[80] Sun F., Zhou Q. (2008). Oxidative Stress Biomarkers of the Polychaete *Nereis diversicolor* Exposed to Cadmium and Petroleum Hydrocarbons. Ecotoxicol. Environ. Saf..

[81] Dias M., Ferreira A., Gouveia R., Madeira C., Jogee N., Cabral H., Diniz M., Vinagre C. (2019). Long-Term Exposure to Increasing Temperatures on Scleractinian Coral Fragments Reveals Oxidative Stress. Mar. Environ. Res..

[82] Valente P., Cardoso P., Giménez V., Silva M.S.S., Sá C., Figueira E., Pires A. (2022). Biochemical and Behavioural Alterations Induced by Arsenic and Temperature in *Hediste diversicolor* of Different Growth Stages. Int. J. Environ. Res. Public. Health.

[83] Robillard S., Beauchamp G., Laulier M. (2003). The Role of Abiotic Factors and Pesticide Levels on Enzymatic Activity in the Freshwater Mussel *Anodonta cygnea* at Three Different Exposure Sites. Comp. Biochem. Physiol. Toxicol. Pharmacol. CBP.

[84] Domingues I., Agra A.R., Monaghan K., Soares A.M.V.M., Nogueira A.J.A. (2010). Cholinesterase and Glutathione-S-Transferase Activities in Freshwater Invertebrates as Biomarkers to Assess Pesticide Contamination. Environ. Toxicol. Chem..

[85] Park J.C., Hagiwara A., Park H.G., Lee J.-S. (2020). The Glutathione *S*-Transferase Genes in Marine Rotifers and Copepods: Identification of GSTs and Applications for Ecotoxicological Studies. Mar. Pollut. Bull..

[86] Park J.C., Kim D.-H., Lee M.-C., Han J., Kim H.-J., Hagiwara A., Hwang U.-K., Park H.G., Lee J.-S. (2018). Genome-Wide Identification of the Entire 90 Glutathione *S*-Transferase (GST) Subfamily Genes in Four Rotifer *Brachionus* Species and Transcriptional Modulation in Response to Endocrine Disrupting Chemicals. Comp. Biochem. Physiol. Part D Genom. Proteom..

[87] King O.C., van de Merwe J.P., Campbell M.D., Smith R.A., Warne M.S.J., Brown C.J. (2022). Interactions Among Multiple Stressors Vary with Exposure Duration and Biological Response. Proc. R. Soc. B Biol. Sci..

[88] Dias M., Madeira C., Jogee N., Ferreira A., Gouveia R., Cabral H., Diniz M., Vinagre C. (2019). Oxidative Stress on Scleractinian Coral Fragments Following Exposure to High Temperature and Low Salinity. Ecol. Indic..

[89] Li X. (2013). Solvent Effects and Improvements in the Deoxyribose Degradation Assay for Hydroxyl Radical-Scavenging. Food Chem..

[90] Sanmartín-Suárez C., Soto-Otero R., Sánchez-Sellero I., Méndez-Álvarez E. (2011). Antioxidant Properties of Dimethyl Sulfoxide and its Viability as a Solvent in the Evaluation of Neuroprotective Antioxidants. J. Pharmacol. Toxicol. Methods.

[91] Ait Alla A., Mouneyrac C., Durou C., Moukrim A., Pellerin J. (2006). Tolerance and Biomarkers as Useful Tools for Assessing Environmental Quality in the Oued Souss Estuary (Bay of Agadir, Morocco). Comp. Biochem. Physiol. Toxicol. Pharmacol. CBP.

[92] Durou C., Poirier L., Amiard J.-C., Budzinski H., Gnassia-Barelli M., Lemenach K., Peluhet L., Mouneyrac C., Roméo M., Amiard-Triquet C. (2007). Biomonitoring in a Clean and a Multi-Contaminated Estuary Based on Biomarkers and Chemical Analyses in the Endobenthic Worm *Nereis diversicolor*. Environ. Pollut..

[93] Fossi Tankoua O., Buffet P.E., Amiard J.C., Amiard-Triquet C., Méléder V., Gillet P., Mouneyrac C., Berthet B. (2012). Intersite Variations of a Battery of Biomarkers at Different Levels of Biological Organisation in the Estuarine Endobenthic Worm *Nereis diversicolor* (Polychaeta, Nereididae). Aquat. Toxicol..

[94] Díaz-Jaramillo M., Martins da Rocha A., Gomes V., Bianchini A., Monserrat J.M., Sáez K., Barra R. (2011). Multibiomarker Approach at Different Organization Levels in the Estuarine *Perinereis gualpensis* (Polychaeta; Nereididae) Under Chronic and Acute pollution Conditions. Sci. Total Environ..

[95] Bastos F.F., Hauser-Davis R.A., Tobar S.A.L., Campos R.C., Ziolli R.L., Bastos V.L.F.C., Bastos J.C. (2013). Enzymatic GST Levels and Overall Health of Mullets from Contaminated Brazilian Lagoons. Aquat. Toxicol..

[96] European Commission Directive 2008/56/EC of the European Parliament and of the Council of 17 June 2008 Establishing a Framework for Community Action in the Field of Marine Environmental Policy (Marine Strategy Framework Directive) 2008. https://www.bsbd.bg/UserFiles/Dir2008-56-EO-EN.pdf.

[97] Pokhrel P., Suzuki J., Fujita M. (2022). Integrated Biomarker Responses of a Brackish Water Clam to Global Warming Conditions. J. Water Environ. Technol..

[98] Mesquita A.F., Gonçalves F.J.M., Gonçalves A.M.M. (2023). Effects of Inorganic and Organic Pollutants on the Biomarkers’ Response of Cerastoderma Edule under Temperature Scenarios. Antioxidants.

[99] Alves A.V., Gusso-Choueri P.K., Altafim G.L., Ferraz M.A., Trevizani T.H., Felix C.S.A., Figueira R.C.L., Abessa D.M.d.S., Choueri R.B. (2025). Influence of CO2-Induced Acidification and Temperature Increased on the Toxicity of Metals in Sediment in the Mussel *Mytella charruana*. Front. Ocean Sustain..

[100] Baag S., Mahapatra S., Mandal S. (2021). An Integrated and Multibiomarker Approach to Delineate Oxidative Stress Status of *Bellamya bengalensis* under the Interactions of Elevated Temperature and Chlorpyrifos Contamination. Chemosphere.

[101] Dias M., Pousão-Ferreira P., Diniz M.S., Marques A., Rosa R., Anacleto P., Maulvault A.L. (2024). Integrated Multi-Biomarker Responses of Juvenile Zebra Seabream (*Diplodus cervinus*) to Warming and Acidification Conditions. Oceans.

