# Supplementary material for: Combined Climate and Chemical Stressors: How Spatial Variability Shapes the Response of Ficopomatus enigmaticus (Fauvel, 1923) to Dimethyl Sulfoxide (DMSO) and Heatwaves, and What It Means for Ecotoxicology
Source: J Xenobiot. 2025 Nov 1;15(6):181. doi: 10.3390/jox15060181 (PMC12641907; doi:10.3390/jox15060181)
Supplement: Supplementary file 1 [file jox-15-00181-s001.zip › jox-3871560-supplementary.pdf]

Journal of
Xenobiotics

MDPI

Supplementary Materials: Combined Climate and Chemical Stressors: How Spatial Variability Shapes the Response of *Ficopomatus enigmaticus* (Fauvel, 1923) to Dimethyl Sulfoxide (DMSO) and Heatwaves, and What It Means for Ecotoxicology

Verdiana Vellani, Manuela Piccardo, Francesca Provenza, Serena Anselmi, Valentina Pitacco, Lovrenc Lipej, Stanislao Bevilacqua and Monia Renzi

Table S1. Summary of mean ± standard deviation for all biomarkers, grouped by site and treatment conditions.

ID	Treatment	Site	PROT	PROT sd	SOD	SOD sd	GST	GST sd	LPO	LPO sd
CTRL 1	CTRL	A	2543.10127	9.39825469	4.56872038	0.18766815	0.02491116	0.00167347	0.16515381	0
CTRL 2	CTRL	A	2400.6962	12.0834703	3.71563981	0.13404868	0.02207885	0.00092388	0.17615255	0
CTRL 3	CTRL	A	2890.56962	17.4539016	4.98578199	0.08042921	0.02589953	0.00085732	0.14194321	0
DMSO 1	DMSO	A	2455.75949	12.0834703	3.86729858	0.26809736	0.02623577	0.00077874	0.17507927	0
DMSO 2	DMSO	A	2220.31646	12.0834703	3.34597156	0.22788275	0.02792387	1.9344E-05	0.18916807	0
DMSO 3	DMSO	A	2370.31646	6.71303906	3.41232227	0.18766815	0.02728181	0.00275353	0.17310982	0
HW 1	HW	A	2381.70886	1.34260781	3.41232227	0.02680974	0.02951081	0.00220869	0.17349513	0
HW 2	HW	A	1757.97468	2.68521562	3.27962085	0.08042921	0.03184934	0.00294335	0.22209437	0
HW 3	HW	A	2582.97468	6.71303906	3.44075829	0.25469249	0.02416891	0.00097502	0.15881418	0.0053655
DMSO+HW 1	DMSO+HW	A	2664.62025	4.02782344	5.26066351	1.4611306	0.0265919	0.00034271	0.15304307	0.01059079
DMSO+HW 2	DMSO+HW	A	2952.27848	0	3.82938389	0.83110181	0.02202174	0.0009688	0.13606838	0.00394621
DMSO+HW 3	DMSO+HW	A	3173.48101	9.39825469	4.57819905	0.44236064	0.02314501	0.00104774	0.10174683	0.03479625
CTRL 1	CTRL	B	1290.75949	0.23868583	4.40655738	0.03709413	0.01350201	0.00001	0.20536	0.0135238
CTRL 2	CTRL	B	1692.99578	0.17901437	5.20655738	0.01854706	0.01120995	0.00001	0.37170	0.00771588
CTRL 3	CTRL	B	1510.50633	0.17901437	4.23606557	0.05564119	0.01598694	0.00001	0.32881	0.00462962
DMSO 1	DMSO	B	1864.45148	0.26852156	4.19672131	0.05564119	0.00731905	0.00001	0.12868	0.00619108
DMSO 2	DMSO	B	1477.80591	0.35802875	4.92459016	0.04636766	0.01131115	0.00001	0.30851	0.00902124
DMSO 3	DMSO	B	1693.83966	0.17901437	5.0295082	0.04636766	0.01559023	0.00003	0.16151	0.00295713
HW 1	HW	B	1085.16878	0.26852156	4.5442623	0.02782059	0.01833422	0.00003	0.58276	0.01975532
HW 2	HW	B	2036.89873	0.32819302	4.47213115	0.01854706	0.00781952	0.00001	0.24134	0.00465632
HW 3	HW	B	1608.24895	0.32819302	3.63278689	0.05564119	0.00656043	0.00001	0.50110	0.01976532
DMSO+HW 1	DMSO+HW	B	1186.20253	0.41770021	4.13114754	0.03709413	0.00781117	0.00001	0.22844	0.00679314
DMSO+HW 2	DMSO+HW	B	1492.173	0.14917865	3.62622951	0.04636766	0.01181554	0.00002	0.62643	0.00917874
DMSO+HW 3	DMSO+HW	B	1235.7173	0.2088501	4.51147541	0.03709413	0.02017575	0.00002	0.43018	0.02073605

Table S2. Mean values and standard deviations of IBRv2I across sites and treatments.

	Site A	Site A	Site B	Site B
**IBRv2i**	mean	sd	mean	sd
CTRL	2.18907519	1.19101501	2.88838323	0.83768907
DMSO	3.70039146	1.29451198	2.88302623	1.61601524
HW	4.95931591	3.59592393	4.19473824	2.71054703
DMSO+HW	3.46307589	1.04951691	3.66582423	1.03674043
